# Factors associated with false negative interferon-γ release assay results in patients with tuberculosis: A systematic review with meta-analysis

**DOI:** 10.1038/s41598-020-58459-9

**Published:** 2020-01-31

**Authors:** Mari Yamasue, Kosaku Komiya, Yuko Usagawa, Kenji Umeki, Shin-ichi Nureki, Masaru Ando, Kazufumi Hiramatsu, Hideaki Nagai, Jun-ichi Kadota

**Affiliations:** 1 Department of Respiratory Medicine and Infectious Diseases, Oita University Faculty of Medicine, 1-1 Idaigaoka, Hasama-machi, Yufu, Oita 879-5593 Japan; 2 Center for Pulmonary Diseases, National Hospital Organization Tokyo National Hospital, 3-1-1 Takeoka, Kiyose, Tokyo 204-8585 Japan

**Keywords:** Tuberculosis, Risk factors

## Abstract

Which factors are related to false negative results of the interferon-γ release assay (IGRA) is unclear. This systematic review described the risk factors associated with false negative IGRA results. Two authors independently identified studies designed to evaluate risk factors for false negative IGRA results from PubMed, the Cochrane Register of Control Trial database, and EMBASE, accessed on October 22, 2018. Meta-analyses were conducted with random-effect models, and heterogeneity was calculated with the *I*^2^ method. Of 1,377 titles and abstracts screened, 47 full texts were selected for review, and we finally included 17 studies in this systematic review. The most commonly studied risk factor (14 studies) was advanced age, followed by low peripheral lymphocyte counts (7 studies), and these factors were associated with false negative results even with different tuberculosis incidences (pooled odds ratio 2.06; 95% CI, 1.68–2.52 in advanced age and 2.68; 95% CI, 2.00–3.61 in low peripheral lymphocyte counts). Advanced age and low peripheral lymphocyte counts may be common risk factors for false negative IGRA results, suggesting that people with these factors need to be carefully followed, even if they have negative IGRA results.

## Introduction

Tuberculosis (TB) is one of the most serious infectious diseases contributing to worldwide morbidity and mortality^1^. The early diagnosis and treatment are effective strategies for preventing the spread of *Mycobacterium tuberculosis* infection. The *M. tuberculosis*-specific interferon-γ release assay (IGRA) is widely considered to identify latent TB infection (LTBI) or to support the diagnosis of active TB infection as an adjunctive test^2–4^. Several risk factors for LTBI and the development of active TB are mentioned in the World Health Organization (WHO) guidelines. HIV infection, contacts bacteriologically confirmed pulmonary TB, initiating anti-TNF treatment, dialysis, organ or hematological transplant and silicosis carry a particularly high risk of TB infection^5^. Screening for LTBI in people with these factors is therefore strongly recommended to prevent the spread of TB infection.

The QuantiFERON Gold in-tube test (QFT-GIT) (Qiagen, Dusseldorf, Germany) as an enzyme-linked immunosorbent assay and T-SPOT.TB test (Oxford Immunotec, Oxford, UK) using an enzyme-linked immune spot (ELISPOT) method are mainly used as commercially available IGRAs. As there is no gold standard for the diagnosis of LTBI, the diagnostic accuracy has been studied using active TB cases. Nevertheless, the accuracy has not reached an adequate level yet. In fact, the pooled sensitivity of these assays for the diagnosis of culture-confirmed active TB has been reported to be 81% and 92% in QFT-GIT and T-SPOT, respectively, and approximately 8–19% patients have negative IGRA results^4^.

False negative results prompt physicians to inappropriately end follow-up and abandon the consideration of prophylactic treatments in patients with possible LTBI. Therefore, analyzing the risk factors for false negative IGRA results is vital to identify patients who need careful follow-up despite negative results. Several risk factors for false negative IGRA results have been reported^6–8^, but there has been no review of these factors.

The present systematic review therefore assessed the risk factors associated with false negative IGRA results using published studies.

## Methods

### Search criteria

This systematic review was conducted according to the guidelines of the preferred reporting items for systematic reviews and meta-analyses (PRISMA) statement and Meta-analysis of observational Studies in epidemiology (MOOSE) guidelines^9,10^. Studies that evaluated the risk factors influencing false negative IGRAs results in patients with bacteriologically confirmed active TB were included. Due to the fact that risk factors for false negative IGRA results are confounded by other variables, we restricted inclusion to studies that performed statistical adjustments by a multivariate analysis in order to exclude low-quality studies.

We searched for studies using PubMed, Cochrane Central Register of Controlled Trials (CENTRAL) and the EMBASE database from August 1992 to October 2018. Combinations of the following search terms were applied: “positivity OR false negative” AND “interferon-gamma release assay OR ELISPOT OR QuantiFERON” (assessed on October 22, 2018). Publications written in languages other than English, studies published only in abstract form and studies in which active TB was not diagnosed by culture positivity, the results of two IGRAs were not described separately or statistical methods were not clearly stated were excluded. We also excluded the studies performed only in children.

The title, abstracts and full texts articles were screened and further evaluated by two authors (YM and KK) independently. Disagreements were resolved by the decision of a third reviewer (JK).

### Data extraction

We extracted the following information from the included studies: study design, sample size, country in which the study was conducted, enrolled age groups, types of IGRAs (e.g. QuantiFERON or/and ELISPOT), single or repeated IGRA testing, where the assay was performed (in-house or commercially) and history of TB and assessed the risk factors for false negative IGRAs results. Regardless of the significance of the results, potential risk factors for false negative IGRA results assessed in two or more studies were analyzed in this review.

We classified the countries where the studies were conducted according to the incidence of TB. The classification proposed by the WHO was used: low-incidence country, incidence of TB <10 new patients per 100,000 population each year; middle-incidence country, incidence of TB 10–100 new patients per 100,000 population each year; and high-incidence country, incidence of TB >100 new patients per 100,000 population each year^1^.

### Assessing the risk of bias

The risk of bias was assessed according to the recommendations outlined in the Cochrane handbook for systematic reviews of interventions version 5.1.0 and MOOSE guidelines for the following items: selection, performance, detection, attrition and publication bias. Each study included in this systematic review was assessed for the quality based on biases using the modified Hayden’s criteria^11^. We assessed the studies for the six factors related to potential biases, as follows: (1) study sample (e.g. source population clearly defined, study population described, and study population represents source population or population of interest), (2) participation rate, (3) analytical procedure clearly described, (4) outcome measurement (e.g. outcome defined and measured appropriately), (5) confounding measurement and accounting (e.g. confounders defined and measured as well as accounted for) and (6) analysis (e.g. analyses described, appropriate and provides sufficient presentation of data). Disagreements between the investigators were resolved by a review of the assessments to reach consensus.

### Data analyses

Meta-analyses were conducted for outcomes with more than two raw data pools available from the included studies. Outcomes were pooled using Mantel-Haenszel risk ratios, and the precision of the estimates was expressed as the 95% confidence interval (CI). Statistical heterogeneity was assessed using the Higgins *I*^2^ tests. A random-effects model was used when significant heterogeneity was found. Publication bias was assessed by an examination of funnel plots^12^. Statistical significance was defined by a p-value < 0.05 for all analyses. The meta-analysis was performed with the Review Manager software program, ver. 5.3 (The Nordic Cochrane Centre, The Cochrane Collaboration, London).

## Results

### Database search and characteristics of the included studies

**Figure 1:**
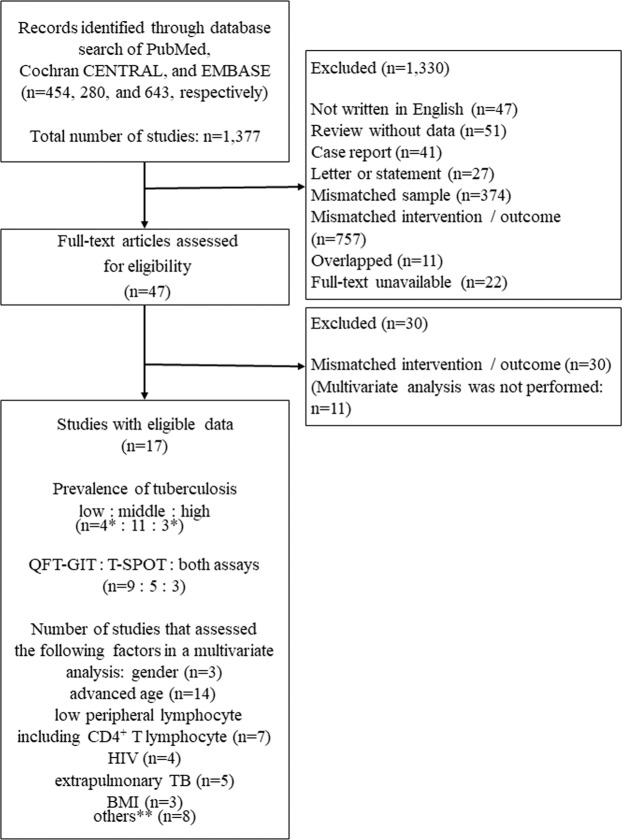
Flow diagram of the study selection. *Eighteen studies are not shown because the results from Denmark (low-incidence country) and Tanzania (high-incidence country) were reported collectively in a single study, so the total number of studies was 17. **Smoking, hospitalization, immunosuppressive conditions, immunosuppressive therapy, malignancy, CRP, low albumin, and HLA type (DRB1*0701 alleles): n = 1.

We identified 454, 280 and 643 studies through PubMed, the CENTRAL and the EMBASE, respectively. We excluded 1,330 studies as the abstract did not meet the inclusion criteria. We excluded 30 of the remaining 47 records after retrieving and inspecting the full text (Fig. 1). We finally included 17 studies in this systematic review: 14^6,7,13–24^ and 3 studies^8,25,26^ were retrospective and prospective observational studies, respectively. These published studies, 4 from low-incidence countries: United States of America (n = 2)^6,22^, Denmark (n = 1)^13^ and the EU (n = 1)^14^; 11 from middle-incidence countries: South Korea (n = 6)^14,16–18,20,26^ and China (n = 4)^8,13,15,21^ and Japan (n = 1)^7^; and 3 from high-incidence countries: Viet Nam (n = 1)^25^, Tanzania (n = 1)^13^ and Zambia (n = 1)^23^. The results from Denmark and Tanzania were reported collectively in one study^13^. Six studies evaluated risk factors for false negative results of IGRAs in a non-HIV population^7,8,16,18,20,24^ (Table 1). How many times the IGRA was performed per sample was not mentioned in any study included in this review.

**Table 1:** Characteristics of the studies included in this systematic review.

Author, year	Nationality	Study design	Sample size	Age, years median (range, IQR or ± SD)	Male (%)	HIV (%)	EPTB (%)	History of TB (%)	IGRA	True-positive (%)	False-negative (%)
Kim 2018	South Korea	retrospective	163	55 (65 < 35%)	85 (52.1)	1 (0.6)	163 (100)	18 (11.0)	QFT-GIT	69.9	28.8
Yang 2018	China	retrospective	2,425	43.6 ± 18.5	1,561 (64)	0	143 (5.9)	nd	T-SPOT	75.1	24.9
Nugyen 2018	USA	retrospective	1,487	47 (IQR: 30–61)	942 (63.3)	90 (13.2)	196 (13.2)	32 (2.2)	875 (65.4) in QTF-GIT 463 (34.6) in T-SPOT	87.7 in total nd in QFT-GIT nd in T-SPOT	12.3 in total 12.2 in QFT-GIT 16.4 in T-SOPT
Di 2018	China	retrospective	98	nd (<30 21.4%, 30–60 50.0%, 60 < 28.6%)	55 (56.1)	nd	69 (70.4)	0	T-SPOT	83.7	16.3
Lian 2017	China	retrospective	556	44.2 (range 0.75–85)	333 (59.9)	2 (0.4)	358 (64.4)	nd	T-SPOT	86.2	13.8
Kown 2015	South Korea	retrospective	1,264	50.3 (IQR: 35–69)	718 (56.8)	0	158 (12.5)	165 (13.1)	QFT-GIT	85.6	14.4
Choi 2015	USA	retrospective	300	48.1 ± 22.1	195 (65.0)	18 (6)	52 (17.3)	nd	QFT-GIT QFT-2G	70.3	29.7
Visser 2015	Europe	retrospective	664	41 (IQR: 30–53) in QFT 41 (IQR: 28–56) in T-SPOT	nd	nd	nd	nd	QTF-GIT T-SPOT	66.6 in total nd in QFT-GIF nd in T-SOPT nd in both IGRA	33.2 in total 22.7 in QFT-GIT 4.8 in T-SPOT 1.0 in both IGRA
Pan 2014	China	prospective	774	45 (range 11–91)	465 (60.1)	0	244 (31.5)	nd	T-SPOT	89.9 in total 91.3 in PTB 86.9 in EPTB	10.1 in total 8.7 in PTB 13.1 in EPTB
Lee 2013	South Korea	prospective	128	65 < 21.1%	53 (41.4)	5 (3.9)	84 (66)	13 (10.2)	T-SPOT	82.8	17
Joen 2013	South Korea	retrospective	168	54.8 ± 20.1	102 (60.7)	0	10 (5.9)	3 (1.8)	QFT-GIT	76.8	23.2
Kim 2013	South Korea	retrospective	44	64 ± 19.0	17 (39)	2 (4.5)	nd	nd	QFT-GIT	68.2	16
Aabye 2012	Denmark Tanzania	retrospective	34 172	50 (range 23–76) 32 (range 15–84)	24 (70.5) 64 (37.2)	4 (8) 75 (43.6)	8 (23.1) nd	0	QFT-GIT	64.7 71.5	11.3 3.8
Hang 2011	Viet Nam	prospective	504	38.8 (IQR: 29.2–50.8)	399 (79.2)	44 (8.7)	0	nd	QFT-GIT	92.3	4.8
Kim 2011	South Korea	retrospective	362	49 (IQR: 16–94)	197 (54.4)	0	0	55 (15.2)	QFT-GIT	85.9	14.1
Komiya 2010	Japan	retrospective	215	67 (IQR: 50–79)	156 (73)	0	0	nd	QFT-G ELISPOT	74 93	23 7.4
Raby 2008	Zambia	retrospective	112	31 (IQR: 25–36)	71 (63)	59 (52.7)	nd	20 (18)	QFT-GIT	74	12

EPTB, extrapulmonary TB; nd, not described.

Among the 17 studies included in our review, the gender, advanced age, low peripheral lymphocyte counts (including CD4^+^ T lymphocytes), HIV positivity, extrapulmonary TB and body mass index (BMI) were studied using a multivariate analysis in 3, 14, 7, 4, 5 and 3 studies, respectively (Fig. 1). None of the 3 reports that studied gender, 5 of the 14 reports that studied advanced age, 4 of the 7 reports that studied low peripheral lymphocyte counts, 3 of the 4 reports that studied HIV positivity, all of the 5 reports^15,19,21,24,26^ that studied extrapulmonary TB and 2 of the 3 reports that studied BMI as risk factors for false negative IGRA results provided the number of patients with false negative results in both the risk group and non-risk group. Other potential risk factors were reported but only in single studies. Therefore, we conducted a meta-analysis of the advanced age, low peripheral lymphocytes counts, HIV positivity, extrapulmonary TB and BMI.

### Assessing the risk of bias

We evaluated the quality in all included studies using the modified Heyden’s criteria (Table 2). The average number that met the six indicators for evaluating the potential bias among the studies was approximately 3.5. While the quality of study participation, analytical procedure, outcome measurement and analysis were mostly good to evaluate among studies, that of the participation rate and the confounding measurement and account were relatively poor. Only 7^14,17,18,20,21,23,25^ of 17 studies had a sufficient participation rate, and the rates in the others were under 50%. For confounding measurement and accounting, eight studies did not assess adequately. The main reason for the low quality in this indicator was that the results of univariate analyses were not described, and possible confounding factors to be evaluated were not considered.

**Table 2:** Quality of studies included in this systematic review.

Author, year	Study sample	Participation rate	Analytical procedure clearly described	Outcome measurement	Confounding measurement and accounting for confounders	Analysis
Kim 2018	**−**	**−**	**+**	**+**	**+**	**+**
Yang 2018	**+**	**−**	**+**	**+**	**−**	**+**
Nugyen 2018	**+**	**−**	nd	nd	**−**	**−**
Di 2018	**+**	**−**	**+**	**+**	**−**	**+**
Lian 2017	**+**	**+**	**+**	**+**	**+**	**+**
Kown 2015	**+**	**+**	**+**	**+**	**+**	**−**
Choi 2015	**+**	**−**	**+**	**+**	**−**	**−**
Visser 2015	**+**	**+**	nd	nd	**−**	**+**
Pan 2014	**+**	**−**	**+**	**+**	**+**	**+**
Lee 2013	**−**	**−**	**+**	**+**	**+**	**+**
Joen 2013	**+**	**−**	**+**	**+**	nd	**−**
Kim 2013	**−**	**+**	**−**	**−**	**+**	**+**
Aabye 2012	**−**	**−**	**+**	**+**	nd	**−**
Hang 2011	**+**	**+**	**+**	**+**	**−**	**+**
Kim 2011	**+**	**+**	**+**	**+**	nd	**−**
Komiya 2010	**+**	**−**	**+**	**+**	**−**	**+**
Raby 2008	**+**	**+**	**+**	**+**	**−**	**+**

+, good assessment; **−**, poor assessment; nd, not described.

**Figure 2:**
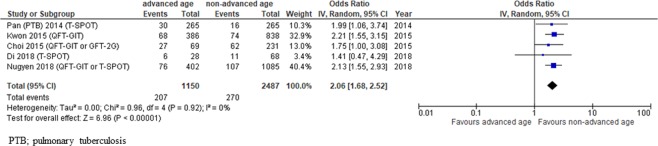
Forest plot for estimating the possibility of advanced age as a risk factor for false negative IGRA results.

**Figure 3:**
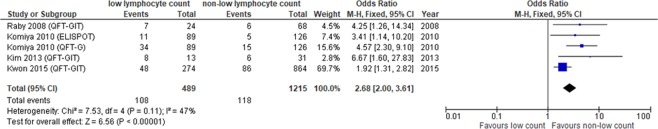
Forest plot for estimating the possibility of low peripheral lymphocyte counts as a risk factor for false negative IGRA results.

**Figure 4:**
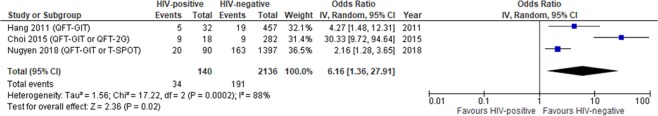
Forest plot for estimating the possibility of HIV positivity as a risk factor for false negative IGRA results.

**Figure 5:**
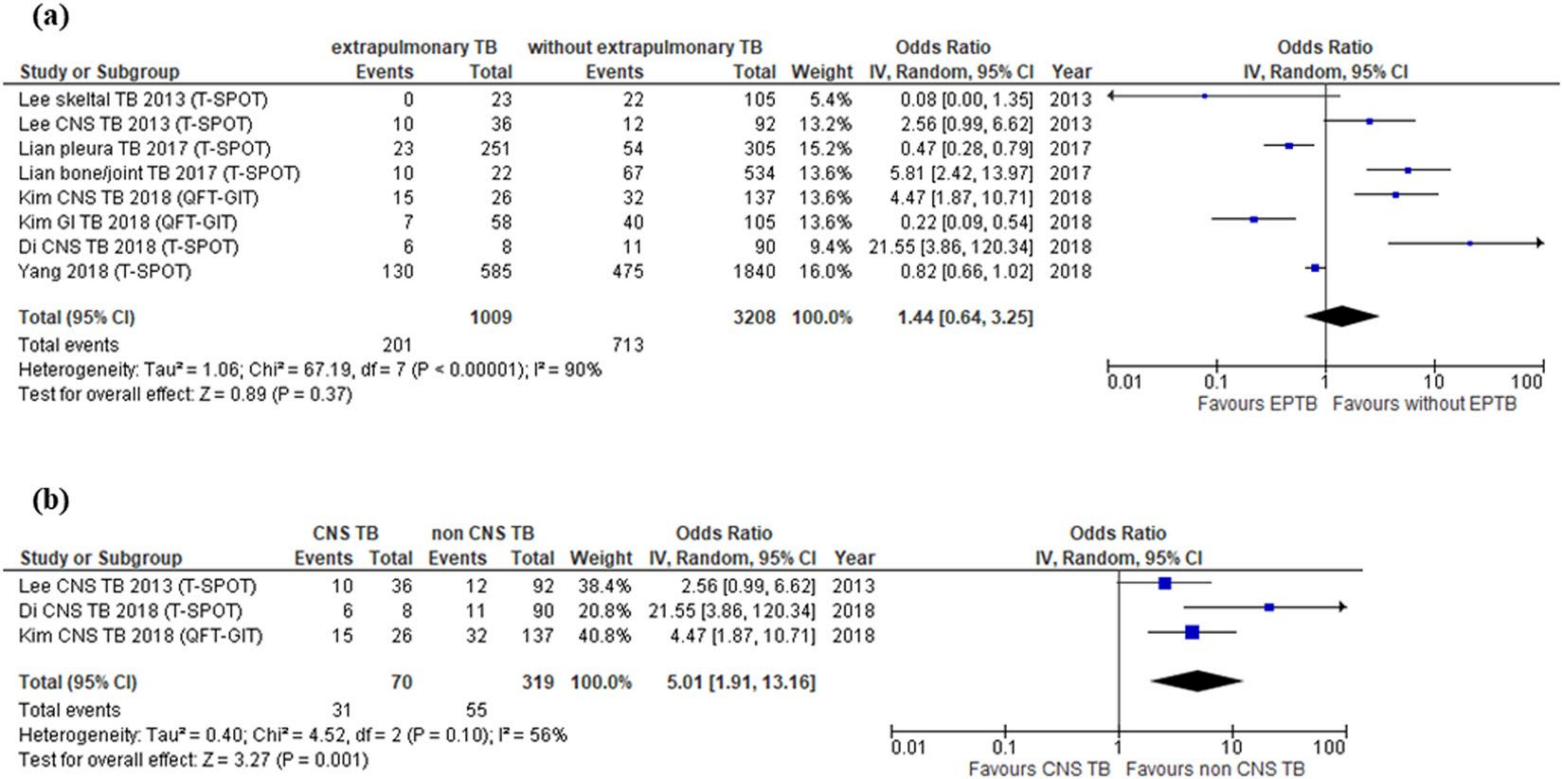
Forest plot for estimating the possibility of extrapulmonary TB as a whole (**a**) and CNS TB specifically (**b**) as risk factors for false negative IGRA results.

**Figure 6:**
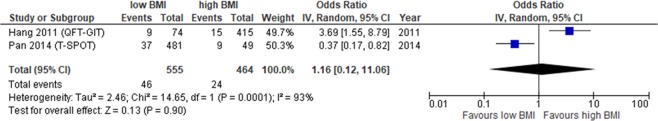
Forest plot for estimating the possibility of the BMI as a risk factor for false negative IGRA results.

High heterogeneity was observed in the studies that evaluated lymphocyte counts, HIV positivity, extrapulmonary TB and BMI as risk factors (Figs. 2–6). As for publication bias, there appeared to be funnel plot asymmetry for low lymphocyte counts, HIV positivity, extrapulmonary TB and BMI but not advanced age, suggesting a low possibility of publication bias (see Supplementary Fig. S1). Due to the small number of studies included in each meta-analysis, Sterne’s test was not appropriate for detecting funnel plot asymmetry^12^.

### Gender

Male gender was studied as a risk factor for false negative results using a multivariate analysis in three studies^7,13,23^. No study showed a significant relationship between male gender and false negative IGRA results (see Supplementary Table S1).

### Advanced age

Pooled odds ratio (pooled OR: 2.06, 95% CI 1.68–2.52) of advanced age calculated with raw data available in 5 of the 14 included studies^6,8,15,20,22^ showed a significant relationship between advanced age and false negative results with very low heterogeneity (Fig. 2). Eight of the 14 studies^8,14,16,20–22,24,25^ found that advanced age was significantly associated with false negative results in a multivariate analysis (see Supplementary Table S2). However, definitions of advanced age varied among the enrolled studies. While Kwon *et al*. compared groups <65 and ≥65 years old^20^, Nugyen *et al*. set 60 years old as the cut-off value^22^. Pan *et al*. used the median age as the cut-off value^8^, and four studies did not clearly state the cut-off value^14,21,24,25^. Joen *et al*. compared the risk of false negative results by age group and showed that the rate increased in the population over 40 years of age^16^.

### Low peripheral lymphocyte counts

Peripheral lymphocyte counts and CD4^+^ T lymphocyte counts were evaluated as a risk factor using a multivariate analysis in five^7,16–18,20^ and two studies^23,24^, respectively; most of the studies were from middle-incidence countries. Low peripheral lymphocyte counts were significantly associated with false negative results in pooled analyses (pooled odds ratio: 2.68, 95% CI 2.00–3.61) using available data from 4 of the 7 studies^7,17,20,23^ with moderate heterogeneity (Fig. 3). Five studies^7,16,17,20,23^ reported that low peripheral lymphocyte counts were significantly associated with false negative results in individual multivariate analyses (see Supplementary Table S3). Three of the five studies performed the analysis in a non-HIV population^7,16,20^, and cut-off values varied among studies^7,16,17,20,23^. Raby *et al*. assessed this factor in the HIV population and reported that a low CD4^+^ lymphocyte count (<200/µl) was a risk factor^23^, whereas Yang *et al*. reported that CD4^+^ lymphocyte counts were not a significant risk factor^24^.

### HIV positivity

Four studies evaluated the association between HIV positivity and false-negative results of IGRA^6,13,22,25^. Two studies were published from countries with a high incidence of both HIV infection and TB^13,25^. In the meta-analysis including raw data available in 3 of the 4 studies^6,22,25^, HIV infection significantly influenced false-negative results of IGRA (pooled OR 6.16, 95% CI 1.36–27.91) with high heterogeneity (Fig. 4). Despite the pooling effect of the meta-analysis, the sample size was small in these studies, creating a large CI and thus limiting their significance. Studies from the United States^6,22^ and Viet Nam^25^ showed that HIV positivity was a significant risk factor in each multivariate analysis (see Supplementary Table S4).

### Extrapulmonary TB

Five studies evaluated the influence of extrapulmonary TB on false negative results of IGRA^15,19,21,24,26^. QFT-GIT and T-SPOT were used for the evaluation of TB in one^19^ and four^15,21,24,26^ studies, respectively. Four^15,21,24,26^ studies reported that extrapulmonary TB was a significant risk factor, and all of these studies conducted assessments by T-SPOT (see Supplementary Table S5). The meta-analysis including 8 data sets from 5 studies^15,19,21,24,26^ in which all types of extrapulmonary TB were evaluated showed that extrapulmonary TB was not a significant risk factor (pooled OR 1.44, 95% CI 0.64–3.25) (Fig. 5a). However, when data were restricted to central nervous system (CNS) TB cases, extrapulmonary TB was indeed significantly associated with false negative results (pooled OR 5.01, 95% CI 1.91–13.16) with moderate heterogeneity (Fig. 5b). The significance of the pooled results was limited because the studies used for this analysis included a small population. In the multivariate analyses of each study, bone or joint TB, pleural TB and CNS TB were reported to be significant risk factors even after adjusting for other variables^15,26^.

### BMI

The BMI was studied as a risk factor for false negative results in 3 studies^8,23,25^. A meta-analysis using available raw data from 2 studies^8,25^ showed that the BMI was not related to false negative results (pooled OR 1.16, 95% CI 0.12–11.06) (Fig. 6). Two studies^8,25^ reported the significant influence of the BMI on false negative results of IGRAs in a multivariate analysis (see Supplementary Table S6). While Pan *et al*. found that a high BMI was significantly associated with false negative results^8^, Hang *et al*. reported the opposite finding in their multivariate analysis^25^.

### Other factors

Smoking^13^, hospitalization (>6 months)^8^, immunosuppressive conditions^18^, immunosuppressive therapy^7^, malignancy^20^, high CRP^16^, low serum albumin (<3.3 mg/dl)^18^ and HLA type (DRB1*0701 alleles)^25^ were reported to be significant risk factors of false negative IGRA results. Although these factors were all assessed in two or more studies, statistical significance was found in only one study (see Supplementary Table S7).

Immunosuppressive conditions as a risk factor were assessed in 11 studies^6,8,14,16–18,21,22,24–26^. However, “immunosuppressive condition” was not clearly defined in most of the studies. Only one study^18^ reported an immunosuppressive condition as a significant risk factor for false negative QFT-GIT results^18^. Komiya *et al*. reported immunosuppressive therapy as a risk factor for false-negative ELISPOT results but did not describe the details of the treatment^7^. Kwon *et al*. reported malignancy as a risk factor for false negative results of QFT-GIT, but they did not describe which type of malignancy and whether or not the patients had received chemotherapy and radiotherapy^20^.

Two studies reported a low serum albumin level (<3.3 mg/dl)^7^ or a longer duration of illness before hospitalization due to TB (>6 months)^8^ as risk factors related to false negative results. Hang *et al*. identified a specific HLA class II allele (DRB1*0701 allele) and reported an increased number of this allele as a risk factor for false negative results of QFT-GIT^25^.

## Discussion

This systematic review revealed that a variety of risk factors influencing false negative results have been reported. The most commonly studied risk factor was advanced age, followed by low peripheral lymphocyte counts, and these factors were significantly associated with false negative results, regardless of TB incidence. HIV positivity and CNS TB were also likely to increase the risk of false negative results, despite being mentioned in only a limited number of studies.

Both an advanced age and low peripheral lymphocyte counts were proposed as significant risk factors for false negative results in two studies, as shown in Supplemental Tables 2 and 3^16,20^. However, whether or not an advanced age and low peripheral lymphocyte count are risk factors independent of each other remains unclear. Two studies using T-SPOT with optimization of the number of lymphocytes per well demonstrated that advanced age but not a low peripheral lymphocyte count was a significant factor^8,21^, suggesting that aging directly attenuates IFN-γ production from a single cell as a reaction to specific TB antigens.

Low peripheral lymphocyte counts may be related to advanced age^27,28^. It is reasonable that peripheral lymphocyte counts are positively correlated with the amount of IFN-γ production in QFT but not T-SPOT, as QFT does not require the optimization of the number of lymphocytes per test. Indeed, Komiya *et al*. reported that the sensitivity of IGRAs depends in part on the peripheral lymphocyte count, and ELISPOT was superior to QFT for detecting TB under low-lymphocyte-count conditions^7^. For elderly patients with low peripheral lymphocyte counts, T-SPOT may be superior for achieving a reduced rate of false negative results. However, whether or not lymphocyte counts were a risk factor for false negative results was mainly evaluated in middle-incidence countries. Studies focusing on peripheral lymphocyte counts are needed in order to verify these results in low- or high-incidence countries as well.

HIV infects CD4^+^ lymphocytes and reduces the number of cells in the periphery. HIV positivity is an independent risk factor for false negative IGRA results based on total peripheral lymphocyte counts, according to the study results of Hang *et al*.^25^. They found no difference in the peripheral lymphocyte counts between the positive QFT-GIT group and the false negative group. However, there is no evidence concerning whether or not HIV positivity is independent of the CD4^+^ lymphocyte counts.

Some studies have suggested that the site of TB infection may significantly affect the T-SPOT sensitivity^14,29,30^. The infection site may therefore also be associated with false negative results of IGRA. While the exact reason why the T-cell response of IGRA differs among organs is unclear, CNS TB was found to be a significant risk factor for false negative IGRA results in the pooled analysis shown in Fig. 5b. The sensitivity of culture and polymerase chain reaction (PCR) of *M. tuberculosis* from cerebral spinal fluid is very low in patients with TB meningitis^31–33^. Given that the blood brain barrier does not allow the components of bacilli to penetrate to the CNS^33^, the frequency of lymphocytes contacting specific antigens in the CNS may be lower than that in the lung, which may explain the increased rate of false negative IGRA results.

The BMI and serum albumin level are usually considered to reflect the nutritional status. For example, a low BMI may indicate malnutrition or severe wasting disease. These conditions may suppress the systemic immune response^34^ and reduce lymphocyte reactions to TB specific antigens on QFT-GIT. Pan *et al*. reported that a high BMI was a significant risk factor for false negative T-SPOT test^8^, although the reasons for this high false negative rate are not fully discussed in the article. Diabetes mellitus (DM) is often concomitant with a high BMI. Faurholt-Jepsen *et al*. reported that the IFN-γ level of QFT was reduced in DM patients with and without TB^35^. This may explain the relationship between a high BMI and the increased risk of false negative results. However, Pan *et al*. also showed that DM was not a significant risk factor in the same population. Another study found that the sensitivity of both T-SPOT and QFT was not affected by DM^36^. The influence of both high BMI and DM on the sensitivity therefore remains controversial.

Immunosuppressive conditions were assessed in 11 studies, and only 1 found significance in a multivariate analysis. Immunosuppressive conditions may be characterized by immunosuppressive therapy, malignancy and likely DM, but no clear definition exists. Therefore, this vague categorization should not be applied for studies assessing the sensitivity of immunological tests; instead, each factor needs to be evaluated separately.

The strength of our study was that we systematically reviewed for the first time the risk factors for false negative results of IGRA with a meta-analysis. However, this systematic review also has several limitations. First, the definitions and cut-off values for each factor varied among the included studies. Common definitions are needed in order to evaluate the impact of each factor more objectively. Second, small-scale studies were included in this systematic review in order to collect as much data as possible. This might generate low heterogeneity, so conducting a large-scale study is encouraged. Finally, whether or not the IGRA was conducted in in-house or commercially, which can cause measurement bias, was unclear in most studies. The IGRA results may be influenced by the handling of samples or the procedures of IGRA testing, in addition to host factors.

This systematic review suggested that advanced age and low peripheral lymphocyte counts were most likely to be associated with false negative IGRA results. Assessments of risk factors for false negative IGRA results with common definitions and the same cut-off values (e.g. advanced age) are needed. Once more reliable evidence is obtained in future studies, the next step will be to create a strategy for reducing false negative results. Substantial variability with repeated IGRA testing was reported, even under identical conditions. This suggests that reversions and conversions around the existing cut-off point should be interpreted with caution^37,38^. Standardization of the assay procedure, including the interval time from blood sampling to the analysis, as well as reconsideration of the best cut-off value by groups with individual risks of false negative IGRA results while considering the reproducibility of the results is required.

## Supplementary information

Supplementary Tables and Figure.
