# Supplementary material for: Factors associated with false negative interferon-γ release assay results in patients with tuberculosis: A systematic review with meta-analysis
Source: Sci Rep. 2020 Jan 31;10:1607. doi: 10.1038/s41598-020-58459-9 (PMC6994686; doi:10.1038/s41598-020-58459-9)
Supplement: Supplementary file 1 — Supplementary Tables and Figure. [file 41598_2020_58459_MOESM1_ESM.pdf]

Factors associated with false negative interferon-γ release assay results in patients with tuberculosis: A systematic review with meta-analysis

Mari Yamasue* 1), Kosaku Komiya* 1), Yuko Usagawa 1), Kenji Umeki 1), Shin-ichi Nureki 1), Masaru Ando 1), Kazufumi Hiramatsu 1), Hideaki Nagai 2), Jun-ichi Kadota 1)

1) Department of Respiratory Medicine and Infectious Diseases, Oita University Faculty of Medicine, 1-1 Idaigaoka, Hasama-machi, Yufu, Oita 879-5593, Japan.

2) Center for Pulmonary Diseases, National Hospital Organization Tokyo National Hospital, 3-1-1 Takeoka, Kiyose, Tokyo 204-8585, Japan.

Supplementary Table S1. Studies evaluating male gender as a risk factor for false-negative IGRA results

author year	rate of male (%)	false-negative (n/N, %)		univariate analysis			multivariate analysis		
		male	female	odds ratio	95% CI	p	odds ratio	95% CI	p
Aabye 2012	109/172 (63.3) in TZ	nd	nd	nd	nd	nd	QFT-GIT 0.99	0.34-2.90	0.99
Komiya 2010	156/215 (73)*	nd	nd	0.751	0.371-1.523	0.428	QFT-G 0.562	0.234-1.351	0.198
				1.054	0.359-3.096	0.924	ELISPOT 0.782	0.215-2.848	0.709
Raby 2008	72/112 (63)	nd	nd	1.69	0.67-3.78	0.268	QFT-GIT 1.1	0.29-3.07	0.861

nd, not described; TZ; Tanzania, * both IGRAs were evaluated collectively and results from each of IGRA were not described

Supplementary Table S2. Studies evaluating advanced age as a risk factor for false-negative IGRA results

author year	cutoff value of age (years)	rate of advanced age (%)	false-negative (n/N, %)		univariate analysis			multivariate analysis		
			advanced age	non-advanced age	odds ratio	95% CI	p	odds ratio	95% CI	p
Yang 2018	older age	nd	nd	nd	1.017	1.012-1.022	< 0.001	T-SPOT 1.018	1.013-1.024	< 0.001
Nugyen 2018	>60	402/1487 (27.0)*	76/402 (18.9)*	107/1085 (9.9)*	2.13*	1.55-2.93*	< 0.001*	QFT-GIT 1.02<br>T-SPOT 1.02	1.01-1.04<br>1.00-1.03	< 0.001<br>0.01
Di 2018	<30	21/ 96 (21.8)	nd	nd	0.188	0.023-1.505	0.115	T-SPOT 0.29	0.035-2.41	0.252
	30-60	47/96 (49.0)	nd	nd	1.66	0.575-4.798	0.349			
	>60	28/96 (29.2)	6/28 (21.4)	11/68 (16.2)	1.438	0.474-4.36	0.521			
Lian 2017	older age	nd	nd	nd	0.983	0.971-0.995	0.006	T-SPOT 0.985	0.972-0.997	0.015
Kwon 2015	>65	386/1224 (31.3)	68/386 (17.6)	74/838 (8.8)	2.21	1.55-3.15	< 0.001	QFT-GIT 1.6	1.05-2.43	0.029
Choi 2015	>65	69/300 (23)	27/69 (39.1)	62/231 (26.8)	1.81	1.03-3.18	0.04	QFT-GIT 1.86	0.98-3.52	0.06
Visser 2015	older age	nd	nd	nd	1.02<br>1.02	1.01-1.03<br>0.99-1.05	< 0.0001<br>0.06	QFT-GIT 1.04	1.02-1.07	< 0.001
Pan 2014	PTB >48.5	265/530 (50)	30/265 (11.3)	16/265 (6.0)	nd	nd	nd	T-SPOT 2.26	1.11-4.58	0.031
	EPTB >39	123/244 (50.4)	22/123 (17.9)	10/121 (8.3)	nd	nd	nd	T-SPOT 2.42	1.09-5.35	0.026
Joen 2013	>40s	nd	nd	nd	nd	nd	nd	QFT-GIT 1.03	1.005-1.056	0.02
Aabye 2012	older age?	nd in TZ	nd	nd	nd	nd	nd	QFT-GIT 0.99	0.95-1.03	0.60
Hang 2011	older age	nd	nd	nd	1.04	1.01-1.06	< 0.05	QFT-GIT 1.04	1.01-1.07	< 0.05
Kim 2011	older age	nd	nd	nd	nd	nd	nd	QFT-GIT 1.00	0.98-1.02	0.91
Komiya 2010	>67	nd	nd	nd	0.591<br>0.612	0.320-1.090<br>0.228-1.641	0.092<br>0.329	QFT-G 1.18<br>ELISPOT 1.76	0.511-2.721<br>0.447-6.934	0.699<br>0.478
Raby 2008	>31	nd	nd	nd	1.20	0.51-2.81	0.672	QFT-GIT 1.13	0.41-3.10	0.810

PTB, pulmonary TB; EPTB, extrapulmonary TB; nd, not described; ?, cutoff value or definition were not shown; TZ, Tanzania

* both IGRAs were evaluated collectively and results from each of IGRA were not described

Supplementary Table S3. Studies evaluating low peripheral lymphocyte count as a risk factor for false-negative IGRA results

author year	cutoff value of lymphocyte count (/µl)	rate of low lymphocyte count (%)	false-negative (n/N, %)		univariate analysis			multivariate analysis		
			low lymphocyte	non-low lymphocyte	odds ratio	95% CI	p	odds ratio	95% CI	p
total peripheral lymphocytes										
Kwon 2015	< 1000	274/1,138 (24.1)	48/274 (17.5)	86/864 (10.0)	1.93	1.32-2.83	0.001	QFT-GIT 1.91	1.24-2.95	0.003
Joen 2013	low lymphocyte	nd	nd	nd	nd	nd	nd	QFT-GIT 0.998	0.998-1.0	0.046
Kim 2013	< 500	13/44 (30)	8/13 (61.5)	6/31 (19.4)	6.667	1.597-27.833	0.009	QFT-GIT 6.017	1.128-32.11	0.036
Kim 2011	low lymphocyte	nd	nd	nd	nd	nd	nd	QFT-GIT 1.00	1.00-1.00	0.83
Komiya 2010	< 1000	89/215 (41.4)	34/89 (38.2)	15/126 (11.9)	5.167	2.679-9.967	< 0.0001	QFT-G 3.778	1.168-7.708	< 0.0001
			11/89 (12.4)	5/126 (4.0)	4.133	1.418-12.045	0.009	ELISPOT 3.314	1.110-9.990	0.033
$CD4^+$ T lymphocytes										
Yang 2018	low lymphocyte?	nd	nd	nd	1.789	1.121-2.856	0.015	T-SPOT 0.423	0.077-2.326	ns
Raby 2008	< 200	24/96 (25)	7/24 (29.2)	6/68 (8.8)	4.71	1.87-11.83	0.001	QFT-GIT 4.68	1.74-12.6	0.002

nd, not described; ?, cutoff value or definition were not shown; ns, not significant

Supplementary Table S4. Studies evaluating HIV positivity as a risk factor for false-negative IGRA results

author year	HIV positivity rate (%)	false-negative (n/N, %)		univariate analysis			multivariate analysis		
		HIV positive	HIV negative	odds ratio	95% CI	p	odds ratio	95% CI	p
Nugyen 2018	90/1,487 (6.1)*	20/90 (22.2)	163/1,397 (11.7)	2.23*	1.32-3.78*	0.003*	QFT-GIT 3.59	1.6-4.76	< 0.001
							T-SPOT 1.34	0.44-4.07	0.6
Choi 2015	18/300 (6)	9/18 (50)	9/282 (3.2)	2.50	0.95-6.57	0.06	QFT-GIT/2G 2.79	1.02-7.61	0.04
Aabye 2012	75/172 (43.6) in TZ	nd	nd	nd	nd	nd	QFT-GIT 2.11	0.73-6.14	0.17
Hang 2011	32/489 (6.5)	5/32 (15.6)	19/457 (4.2)	4.26	1.48-12.28	< 0.05	QFT-GIT 4.26	1.48-12.28	< 0.05

nd, not described; TZ, Tanzania

* both IGRAs were evaluated collectively and results from each IGRA were not described

Supplementary Table S5. Studies evaluating extrapulmonary tuberculosis as a risk factor for false-negative IGRA results

author year	infection site of TB	rate of EPTB (%)	false-negative (n/N, %)		univariate analysis			multivariate analysis		
			EPTB	without EPTB	odds ratio	95% CI	p	odds ratio	95% CI	p
Kim 2018	CNS TB	26/163 (15.9)	15/26 (58.3)	32/137 (23.4)	4.38	1.83-10.51	0.001	QFT-GIT 1.16	0.19-1.31	0.16
	GI TB	58/163 (35.5)	7/58 (12.1)	40/105 (38.1)	0.31	0.12-0.75	0.01	QFT-GIT 0.5	0.32-4.19	0.82
Di 2018	CNS TB	8/98 (8.2)	6/8 (75)	11/90 (12.2)	21.273	3.808-118.834	< 0.01	T-SPOT 17.4	3.068-98.671	0.001
Lian 2017	bone/joint TB	22/556 (4.0)	10/22 (45.4)	67/534 (12.5)	0.241	0.096-0.602	0.002	T-SPOT 0.321	0.125-0.823	0.018
	pleura TB	251/556 (45.1)	23/251 (9.2)	54/305 (17.7)	2.252	1.331-3.811	0.002	T-SPOT 1.954	1.139-3.351	0.015
Lee 2013	CNS TB	36/128 (28.1)	10/36 (27.8)	12/92 (13.0)	2.56	0.99-6.62	0.05	T-SPOT 2.56	0.99-6.62	0.05
	skeletal TB	23/128 (18.0)	0/23	22/105 (21.0)	0.17	0.02-1.35	0.09	T-SPOT 0.03	0.03-1.85	0.17
Yang 2018	without EPTB	1840/2425 (75.9)	130/585 (22.2)	475/1840 (25.8)	1.218	0.976-1.519	0.081	T-SPOT 1.291	1.026-1.623	0.029

EPTB, extrapulmonary TB; CNS, central nervous system (including meningitis); GI, gastrointestinal; nd, not described

Supplementary Table S6. Studies evaluating body mass index as a risk factor for false-negative IGRA results

author year	cutoff value of BMI (kg/m^2)	rate of BMI out of cutoff value (%)	false-negative (n/N, %)		univariate analysis			multivariate analysis		
			low BMI	not low BMI	odds ratio	95% CI	p	odds ratio	95% CI	p
Pan 2014	> 25	49/530 (9.2)	37/481 (7.7)	9/49 (18.3)	nd	nd	nd	T-SPOT 2.43	1.05-5.63	< 0.05
Hang 2011	< 16	74/489 (15.1)	9/74 (12.2)	15/415 (3.6)	7.27	2.17-24.38	< 0.05	QFT-GIT 5.42	1.48-19.79	< 0.05
Raby 2008	< 18.5	nd	nd	nd	1.29	0.53-3.18	0.574	QFT-GIT 1.59	0.58-4.38	0.368

BMI, body mass index; nd, not described

Supplementary Table S7. Other significant risk factors for false-negative IGRA results reported in a single study

risk factor	cutoff value or definition of risk factor	false-negative (n/N, %)		multivariate analysis		
author year	rate (%)	with risk factor	without risk factor	odds ratio	95% CI	p
smoking	smoking					
Aabye 2012	19/31 (61.2) in DK	9/19 (47.3)	0/12	QFT-GIT 11.3	1.2-287.7	0.02
	17/145 (11.7) in TZ	6/17 (35.3)	16/112 (14.3)	QFT-GIT 3.8	1.2-11.8	0.02
a longer duration of illness before hospitalization			>6 months			
Pan 2014	122/ 530 (23)	18/122 (14.7)	28/408 (6.8)	T-SPOT 2.46	1.24-4.92	< 0.05
*immunosuppressive conditions**						
Kim 2011	47/362 (13)	16/3 (34.0)	35/315 (11.1)	QFT-GIT 2.98	1.38-6.47	0.006
immunosuppressive therapy						
Komiya 2010	18/215 (8.3)	5/18 (27.8)	11/197 (5.6)	ELISPOT 0.202	0.060-0.684	0.01
malignancy						
Kwon 2015	83/1,224 (6.8)	12/83 (14.5)	130/1141 (11.4)	QFT-GIT 2.5	1.33-4.60	0.004
high CRP	cutoff value was not described					
Joen 2013	nd	nd	nd	QFT-GIT 1.069	1.013-1.217	0.014
low serum albumin	< 3.3 g/dL					
Komiya 2010	nd	nd	nd	QFT-G 2.507	1.200-5.234	0.013
HLA type	DRB1*0701 alleles					
Hang 2011	61/1,008 (6.1)	11/61 (18.0)	37/947 (39.1)	QFT-GIT 5.09	2.31-11.22	< 0.05

DK: Demark, TZ: Tnzania, nd: not described

*Immunosuppressive conditions: 1) solid cancer patients undergoing anticancer chemotherapy or radiotherapy, 2) patients with malignant hematological diseases, 3) organ or bone marrow transplant recipients; 4) patients with end-stage renal disease receiving renal replacement therapy, 5) patients with advanced (Child-Pugh class C) liver cirrhosis, and 6) patients receiving immunosuppressive drug treatment (15 mg/d prednisone or a combination of low-dose corticosteroids and azathioprine, mycophenolate, methotrexate, cyclosporine, or cyclophosphamide.

Supplementary figure S1.

(a)

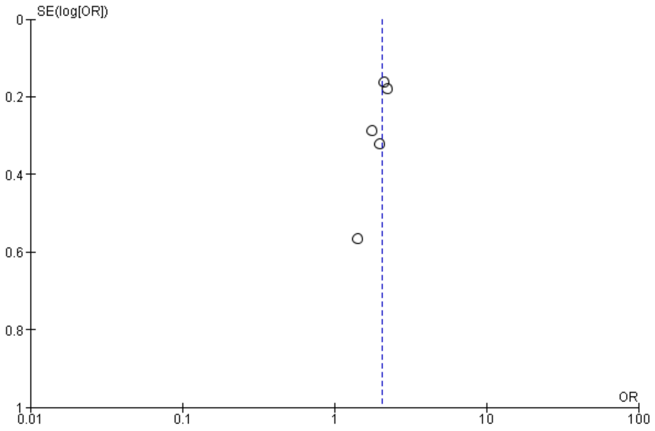

(d)

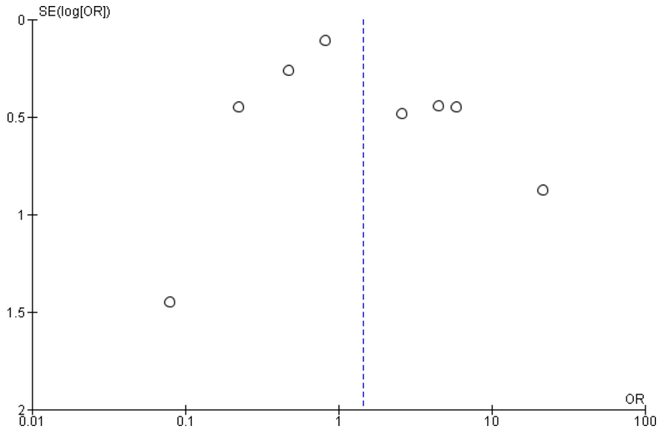

(b)

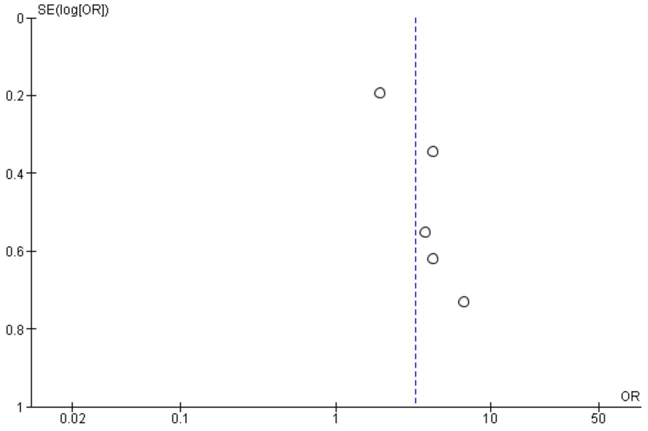

(e)

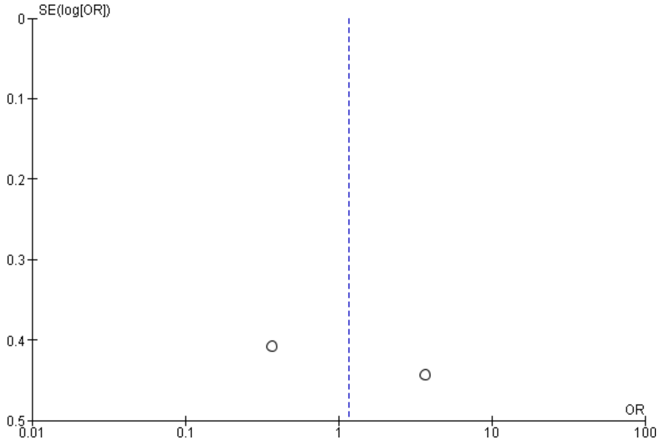

(c)

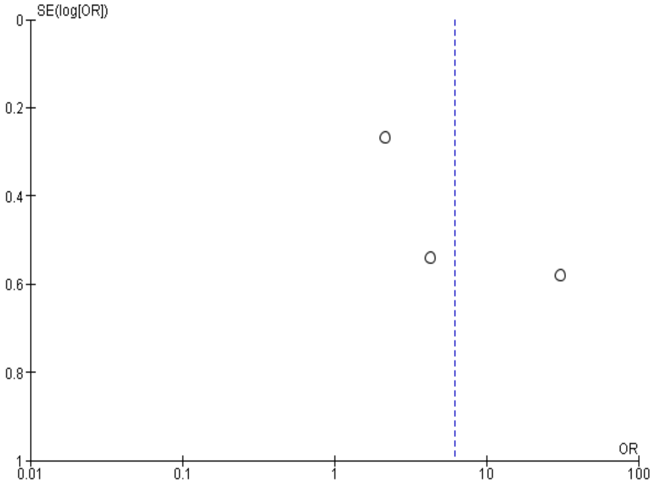

Supplementary figure S1. Funnel plot of studies evaluated advanced age (a), peripheral lymphocyte counts (b), HIV positivity (c), extrapulmonary TB (d) or BMI (e) as a risk factor for false negative results of IGRA.
